# Supplementary material for: From cockroaches to tanks: The same power-mass-speed relation describes both biological and artificial ground-mobile systems
Source: PLoS One. 2021 Apr 26;16(4):e0249066. doi: 10.1371/journal.pone.0249066 (PMC8075212; doi:10.1371/journal.pone.0249066)
Supplement: S1 File — (DOCX) [file pone.0249066.s001.docx]

Supporting Information

# **Biological organisms and sources of related data:**

Cockroaches data are adapted from [1, 2]; spiders [3]; crabs [4]; humans [5, 6, 7, 8, 9]; quail [10, 8]; chipmunks [10, 8]; dogs [10, 8, 9]; kangaroos [8, 9]; horses [9, 11]; elephants [12], frogs [13], geckos [14], other lizards [15], penguin [16], lemurs [17], giant tortoises [18], as well as the kangaroo rat, ground squirrel, spring hare, wild turkey, stump-tailed monkey, greater rhea, and sheep [8].

It should be noted that for measuring mechanical power expended by animals in their locomotion, several classes of technique are used and reported in literature: (a) force-measuring plates or treadmills, (b) video analysis of movement of body and limbs, (c) the combination of the two, and (d) other methods, such as attaching inertial sensors to the body and limbs of an animal. Most techniques and data refer to the work associated with the movements of the center of the mass of the animal, i.e., P_ext_, while a few data point add the movements of the limbs, i.e., P_int_. We use data obtained by any of these techniques, whichever is available in the literature. All of these techniques – with inevitable differences in assumptions – can serve as an order-of-magnitude approximation of P_a_, the amount of mechanical energy produced by the muscles and expended to propel the system, as we show in the Discussion section of the main paper. While different techniques are likely to produce somewhat different measures, the differences are sufficiently small for an allometric study like ours, where the accuracy of the relations tends to be limited to an order of magnitude.

# **Artificial systems and sources of related data:**

Approaches to obtaining or estimating the power, mass, and speed of such systems differed. Unless otherwise specified below, reference [19] presents most of dataset and notes explaining the sources and methods.

In most cases of modern vehicles, the mechanical horsepower of a system was taken as the maximum horsepower specified by the manufacturer or other authority for the engines of the system. For example, for a heavy dump truck, a database of specifications [20] provides the engine horsepower of each truck. The rated power is by far the most readily and consistently available. Typically, in modern practice, ISO 1585 would be used to measure the rated power of engine. In older sources, we assumed that the power quoted was comparable to modern rated power. The mass of the dump truck is the sum of the vehicle’s empty weight plus the weight of its specified maximum load. Similarly, speed is the specified maximum speed. Even with maximum speed and load, the actual mechanical power expended for locomotion may be lower than the rated power. However, as we mentioned earlier, differences are likely to be sufficiently small for an allometric study like ours, where the accuracy of the relations tends to be limited to an order of magnitude.

In some cases, particularly bicycles, UTVs and trains, actual experimental data were available for the maximum speed attainable with the maximum engine power and a given load, or the maximum distance traveled with a given energy supply and a given load.

In the cases of horse-drawn guns, the only source of power are the horses. We estimated the mechanical power as follows. Number of horses per gun were taken from various military history studies (see [21]); the power of a horse was taken from [11] assuming 500 kg horse trotting at 15 km/hr. This is a lower limit of estimate because [11] did not study a trained artillery horse that in addition to propelling itself, also pulls a wheeled gun, amounting typically to about 200-300 kg per horse.

In many of the systems considered in this study, both offroad and on-road speed are specified. In such cases, we preferred to use the offroad speed because it is more relevant to our interests in defense-related systems (which typically operate in offroad environments) and is more consistent with the locomotion of animals, who generally do not move on hard, smooth surfaces like a paved road.

The following systems are included in our dataset:

- Bicycles included both human-powered and electric types; data are based on [22, 23, 24, 25].
- Machine-gun teams comprise machine guns and pertinent equipment, sometimes with wheeled or sled-like carriages, operated and carried by teams of human soldiers, and sometimes pulled by teams of horses. The systems were designed and operated at various times between 1868 and 2015. Because machine-gun teams and horse-towed artillery (described below) are powered by biological movers (humans and horses), these data can be seen as a useful bridge, i.e., as intermediate cases between strictly biological and strictly artificial systems.
- Utility task vehicles (UTVs) are small 2- to 6-person, four-wheel offroad vehicles used for recreation, farming, and other purposes.
- Horse-towed artillery are various artillery systems used between the 16^th^ and early 20^th^ centuries; each system typically includes a cannon along with its carriage, limber, sometimes caisson, team of horses, and a team of soldiers.
- Motor-towed artillery are artillery systems appearing between the 1920s and early 2000s; each system typically includes cannon along with its carriage, a truck or a tractor that pulls the cannon, and a team of soldiers.
- “Technicals” are systems that appeared between the 1970s and 2000s and typically consist of a civilian truck with heavy machine guns or automatic cannons installed in the bed of the truck.
- Trucks Class 1‒4 in our dataset are modern (2010 and beyond) commercial civilian trucks that belong to classes 1 through 4 of the US vehicle classification system (e.g., light pickup trucks, cargo vans, heavy-duty pickups, and city delivery trucks). No vehicles in these classes can exceed a gross weight (i.e., the weight of the loaded vehicle) of 16,000 lb.
- Trucks Class 5‒8 in our dataset are modern (2010 and beyond) commercial civilian trucks that belong to classes 5 through 8 of the US vehicle classification system (e.g., beverage trucks, furniture trucks, tractor-trailer combinations, cement trucks, and dump trucks. Vehicles up to class 7 are limited to a gross weight of 33,000 lb; class 8 includes vehicles with a gross weight over 33,000 lb.
- Armored Fighting Vehicles (AFVs) and Armored Personnel Carriers (APCs) are military vehicles intended mainly for transporting soldiers to and around the battlefield. Examples in our dataset appeared in the 1920s through early 2000s and typically include a machine gun or an automatic cannon, and armor that can protect against machine guns and relatively light weapons.
- Tanks and self-propelled guns (SPGs) are military vehicles armed with heavy guns and often with heavy armor suitable for protecting against powerful weapons. The examples in our dataset appeared between 1916 and 2015.
- Dump trucks in our dataset are modern (post-2010) vehicles used in mining and constructions for carrying large loads of bulk materials. Their gross weight in some cases is far greater than any other types of ground vehicles, approaching 1,000,000 kg.
- Trains included high-speed passenger as well as freight types, data were found in [26, 27, 28, 29, 30, 31, 32, 33].
- NASA Crawler transporter vehicle, data from [34].

# **Explanations of power expenditure relations in biological organisms:**

The literature offers numerous explanations for the diverse allometric relations among mass, body length, speed, and power expenditures of biological organisms. For example, Bejan and Marden [35] theorize that systems evolve in a way that minimizes the destruction of useful energy. This optimization yields the empirically observed relations in which the speed of an animal is proportional to the body mass raised to the power of 1/6. The authors show that these relations hold for running, flying, and swimming.

A number of researchers argue that animal locomotion and scaling are driven, to a large extent, by acceptable stresses in the structural components and muscles of the animals. McMahon [36, 37] offers theoretical models that yield empirically observed power-law relations between an animal’s body mass and metabolic energy or maximum speed. These theoretical models hinge on either maintaining the acceptable static stresses in animals’ muscles and bones or avoiding a limb buckling failure. Others, for example, Biewener and Taylor [38] and Biewener [39] show empirically that the top speed of an animal is linked to the peak strains (or stresses) developed in its limbs, keeping bone and muscle stresses fairly constant across a wide range of mass.

In Iriarte-Diaz [40], the scaling of animals’ maximum relative speed (body lengths per second) is shown to be different among small and large (above approximately 30 kg) mammals. Among small species, the relative speed diminishes modestly with body mass. In large mammals, however, the relative speed is strongly and negatively related to body mass. The author explains these two regimes of the allometric relation are based on the need to maintain the acceptable level of stress in bones.

Meyer-Vernet and Rospars [41] find that for about 460 species of various taxonomic groups, the maximum relative speed is on the order of magnitude of 10 body lengths per second over a wide mass range of running and swimming animals. The authors relate this generic result to basic biological properties, in particular, the relation between muscle specific tension and molecular motors.

In a rare exploration of commonalities between organisms and artificial systems, Marden [42] notes a strong quantitative similarity between biological and engineered motors. Both exhibit at least two regimes for the mass scaling of maximum force output. One scaling regime comprises motors that create slow translational motion with force outputs limited by the axial stress capacity of the motor, which results in maximum force output scaling as motor mass to the power of 0.67. Another scaling regime comprises motors that cycle rapidly, with significant accelerations, and for whom inertia and fatigue life appear to be governing constraints. The Group 1 and 2 scaling relationships (with different exponents in relations of force output to mass) intersect at a motor mass of 4,400 kg, above which all motors are limited by stress. Perhaps it is not a mere coincidence that such motor mass corresponds roughly to vehicle mass of 35,000 kg [42], approximately where we too observe a break in the exponent of relation between power and mass (e.g., Fig. 1 and equation (2)).

# **Factors influencing the speed of a vehicle:**

The following is a sample of the factors involved in the models found experimentally to be necessary for predicting a system’s speed [43, 44, 45]:

- Soil properties: moisture content, sand, frozen, snow, ice, cultivated farm land, urban rubble
- Terrain, road and environment: slopes, curves, ground roughness, obstacles types and shapes, weather, visibility, presence and type of vegetation on the route
- Driver: tolerance to shock and vibration, agility of perception and reaction to road obstacles, acceptance of risk, autonomous controls
- Vehicle features and characteristics: size; geometry; multi-body; center of gravity; tipping angle; wheeled, tracked, or legged; sizes and properties of track, tires, or legs; acceleration and breaking performance; manned or autonomous; suspension; steering.

# **Power requirements of the EDMEC robotic system:**

Complex terrains (dense forests, rocky plains, moderately difficult mountain trails, building rubble) are commonly encountered military operational environments, which many wheeled and tracked vehicles are unable to traverse. A quadruped like EDMEC, on the other hand, can go where the soldier goes, thereby avoiding roads and paths that an adversary can more easily monitor or attack. Quadrupedal systems such Boston Dynamics’s Spot Mini [46] and Ghost Robotic’s Vision60 [47] are already being sold for inspection, security, and payload delivery applications in unstructured terrains.

To estimate EMDEC’s power requirements, we created a reduced-order physics model of the system using the Spring Loaded Inverted Pendulum (SLIP) model for legged locomotion. The SLIP model is a single-leg runner that shows a high degree of similarity to the dynamics, design, and control of legged robots [48]. We tuned the model to achieve a stable gait for the system and then calculated the system power from the forces, torques, and velocities output by the model.

To convert the single-leg SLIP model to a quadruped, we followed the assumptions of ref [49]. The SLIP model approximates that one leg can carry a 300 kg mass with a 4,200 W average total mechanical power. This single leg alternates between a ground contact phase (stance) and an in-air swinging phase (flight). For certain quadruped gaits like trotting or bounding, two legs remain on the ground while the other two swing in the air. To complete a gait cycle, or a time period that contains the sequence of events where we start the locomotion events with one foot (or set of feet in cases of quadrupedal locomotion such as bounding or trotting) contacting the ground and continue counting until that same foot (or set of feet) comes in contact with the ground again. Our analysis assumes that 2 legs can carry a 600 kg mass at the same average total mechanical power as one leg carries a 300 kg mass. To obtain the power output of a full gait cycle, where one pair of legs is in stance and the other pair of legs is in the air, we add the power of the opposite pair of legs to bring the average total mechanical power to 8,400 W for a quadrupedal runner.

This power requirement can be reduced by applying optimizations like power regeneration and passive energy storage elements used in developing the MIT Cheetah robot [50], one of the most efficient legged robots reported to date. Like the Cheetah robot, we assume EMDEC uses springs to conserve 50% of its locomotor power. We also assume that EDMEC is able to regenerate 25% of spring locomotor power through regenerative braking. The remaining power for thrusting and braking is generated by the motors. Today’s robots like the Cheetah lose 75% of their power through heat and friction losses. For EDMEC, we assume that we can improve this to only 50% power loss to heat and friction. With all these assumptions, the total power needed for locomotion is 4,920 W.

# Table of Data

| **Animal/System** | **system mass (kg)** | **mechanical power (hp)** | **speed (km/h)** |
| --- | --- | --- | --- |
| **Animals** |  |  |  |
| American cockroach | 8.10E-04 | 1.63E-07 | 1.62E+00 |
| American cockroach | 8.10E-04 | 8.70E-07 | 2.88E+00 |
| American cockroach | 8.10E-04 | 1.52E-06 | 3.24E+00 |
| American cockroach | 8.10E-04 | 1.85E-06 | 5.40E+00 |
| Cockroach | 2.60E-03 | 2.09E-07 | 3.60E-01 |
| Cockroach | 2.60E-03 | 1.78E-06 | 2.16E+00 |
| Ghost crabs | 3.00E-02 | 8.05E-06 | 7.20E-01 |
| Ghost crabs | 3.00E-02 | 3.62E-05 | 3.24E+00 |
| Ghost crabs | 5.00E-02 | 6.71E-06 | 3.60E-01 |
| Ghost crabs | 5.00E-02 | 8.72E-05 | 4.50E+00 |
| Spider Grammostola mollicoma | 1.85E-02 | 7.05E-07 | 2.02E-01 |
| Spider Grammostola mollicoma | 1.85E-02 | 3.86E-06 | 6.80E-01 |
| Frog Kassina maculate | 8.30E-03 | 1.23E-06 | 3.60E-01 |
| Frog Kassina maculate | 8.30E-03 | 2.34E-06 | 6.84E-01 |
| Gecko Hemidactylus garnotii | 2.50E-03 | 4.93E-06 | 1.01E+00 |
| Gecko Hemidactylus garnotii | 2.50E-03 | 1.16E-05 | 2.70E+00 |
| lizard Coleonyx variegatus | 3.70E-03 | 1.49E-06 | 1.08E+00 |
| lizard Coleonyx variegatus | 3.70E-03 | 4.47E-06 | 2.70E+00 |
| emperor penguin | 2.10E+01 | 1.06E-02 | 9.00E-01 |
| emperor penguin | 2.10E+01 | 5.07E-03 | 2.16E+00 |
| emperor penguin | 2.10E+01 | 2.28E-02 | 3.24E+00 |
| Lemur catta | 2.52E+00 | 2.54E-03 | 1.80E+00 |
| Lemur catta | 2.52E+00 | 2.00E-02 | 6.12E+00 |
| Giant Galápagos tortoises | 1.42E+02 | 9.53E-03 | 3.60E-01 |
| Giant Galápagos tortoises | 1.42E+02 | 1.45E-02 | 6.84E-01 |
| Giant Galápagos tortoises | 1.42E+02 | 1.94E-02 | 1.22E+00 |
| Humans | 6.00E+01 | 1.79E-01 | 8.00E+00 |
| Humans | 1.70E+02 | 5.07E-01 | 8.00E+00 |
| Human | 7.00E+01 | 9.94E-01 | 3.20E+01 |
| Human | 7.00E+01 | 5.95E-01 | 1.62E+01 |
| Painted quail | 4.40E-02 | 8.72E-05 | 1.37E+00 |
| Painted quail | 4.40E-02 | 8.05E-05 | 1.37E+00 |
| Painted quail | 4.40E-02 | 1.56E-04 | 5.47E+00 |
| Painted quail | 4.40E-02 | 1.34E-04 | 5.47E+00 |
| king quail | 4.00E-02 | 1.42E-04 | 5.65E+00 |
| bobwhite quail | 1.80E-01 | 9.98E-04 | 9.47E+00 |
| Chipmunk | 6.10E-02 | 2.03E-04 | 4.25E+00 |
| Chipmunk | 6.10E-02 | 1.76E-04 | 4.25E+00 |
| Chipmunk | 6.10E-02 | 3.80E-04 | 5.76E+00 |
| Chipmunk | 6.10E-02 | 2.52E-04 | 5.76E+00 |
| eastern chipmunk | 1.00E-01 | 5.40E-04 | 1.14E+01 |
| Dog | 5.00E+00 | 2.52E-02 | 1.33E+01 |
| Dog | 5.00E+00 | 1.72E-02 | 1.33E+01 |
| Dog | 1.10E+01 | 1.15E-01 | 2.99E+01 |
| Dog | 2.00E+01 | 5.37E-02 | 5.40E+00 |
| Dog | 2.00E+01 | 1.50E-01 | 1.80E+01 |
| kangaroo rat | 7.00E-02 | 3.08E-04 | 1.04E+01 |
| ground squirrel | 1.90E-01 | 2.94E-04 | 8.82E+00 |
| springhare | 2.50E+00 | 3.16E-02 | 2.40E+01 |
| wild turkey | 7.00E+00 | 6.72E-02 | 1.80E+01 |
| stump-tailed monkey | 3.60E+00 | 5.46E-02 | 2.20E+01 |
| greater rhea | 2.25E+01 | 1.51E-01 | 1.80E+01 |
| red kangaroo | 2.05E+01 | 3.38E-01 | 2.80E+01 |
| red kangaroo | 1.80E+01 | 2.85E-01 | 2.48E+01 |
| red kangaroo | 9.00E+00 | 1.45E-01 | 2.23E+01 |
| Tammar wallaby | 4.50E+00 | 9.40E-02 | 2.16E+01 |
| sheep | 7.30E+01 | 1.67E-01 | 1.25E+01 |
| horse | 2.80E+02 | 8.56E-01 | 7.20E+00 |
| horse | 2.80E+02 | 3.99E+00 | 2.52E+01 |
| horse | 5.15E+02 | 9.68E-01 | 7.20E+00 |
| horse | 5.15E+02 | 5.81E+00 | 2.16E+01 |
| horse | 5.15E+02 | 2.82E+01 | 4.32E+01 |
| Asian Elephant | 2.81E+03 | 5.54E-01 | 2.52E+00 |
| Asian Elephant | 2.81E+03 | 9.35E-01 | 5.58E+00 |
| Asian Elephant | 2.81E+03 | 2.45E+00 | 1.12E+01 |
| Asian Elephant | 2.81E+03 | 4.07E+00 | 1.69E+01 |
| **Bicycles** |  |  |  |
| Mountain bike race | 9.90E+01 | 3.65E-01 | 3.06E+01 |
| recreational cyclist | 1.00E+02 | 1.00E-01 | 1.60E+01 |
| recreational cyclist | 1.00E+02 | 3.00E-01 | 2.96E+01 |
| E-bike | 1.13E+02 | 9.66E-02 | 1.60E+01 |
| E-bike | 1.13E+02 | 1.92E-01 | 2.24E+01 |
| E-bike | 1.13E+02 | 3.97E-01 | 3.20E+01 |
| E-bike | 8.10E+01 | 6.71E-02 | 1.04E+01 |
| E-bike | 8.10E+01 | 1.61E-01 | 1.76E+01 |
| E-bike | 1.74E+02 | 2.01E-01 | 1.04E+01 |
| E-bike | 1.74E+02 | 3.62E-01 | 1.52E+01 |
| **Machine gun teams** |  |  |  |
| Gatling gun 1870, 1.0 caliber | 5.01E+03 | 6.80E+00 | 5.00E+00 |
| Gatling gun 1870, .50 caliber | 2.61E+03 | 3.50E+00 | 5.00E+00 |
| Gatling gun 1870, .45 caliber | 1.69E+03 | 2.40E+00 | 5.00E+00 |
| Hotchkiss Mle 1897 | 6.83E+02 | 7.00E-01 | 3.00E+00 |
| pre-1900 Maxim 45 caliber on artillery-like carriage | 1.34E+03 | 1.70E+00 | 5.00E+00 |
| Skoda 1893 | 6.83E+02 | 7.00E-01 | 3.00E+00 |
| Hotchkiss .303 Mk I | 4.88E+02 | 5.00E-01 | 4.00E+00 |
| Madsen 1904 | 3.90E+02 | 4.00E-01 | 4.00E+00 |
| Schwarzlose 1907 | 6.83E+02 | 7.00E-01 | 3.00E+00 |
| German Maxim MG08, crew 9 | 8.49E+02 | 9.00E-01 | 3.00E+00 |
| German Maxim MG08, crew 4 | 3.94E+02 | 4.00E-01 | 3.00E+00 |
| Hotchkiss Portative Mle1909 | 2.93E+02 | 3.00E-01 | 4.00E+00 |
| Russian PM1910 | 7.49E+02 | 8.00E-01 | 4.00E+00 |
| Vickers Mk1, crew 3 | 2.93E+02 | 3.00E-01 | 3.00E+00 |
| Vickers Mk1, crew 6 | 5.86E+02 | 6.00E-01 | 3.00E+00 |
| Lewis Mk1 | 1.95E+02 | 2.00E-01 | 4.00E+00 |
| German Maxim MG08/15, crew 4 | 3.94E+02 | 4.00E-01 | 4.00E+00 |
| Browning M1917 crew 6 | 5.85E+02 | 6.00E-01 | 3.00E+00 |
| Vickers-Berthier | 2.93E+02 | 3.00E-01 | 3.00E+00 |
| Browning Automatic Rifle M1918A2 | 1.95E+02 | 2.00E-01 | 4.00E+00 |
| Chatellerault Mle 24/29 | 2.93E+02 | 3.00E-01 | 3.00E+00 |
| Bren Mark 1 | 2.93E+02 | 3.00E-01 | 4.00E+00 |
| Fiat 12mm | 5.85E+02 | 6.00E-01 | 2.00E+00 |
| Vickers 12.7mm | 5.85E+02 | 6.00E-01 | 2.00E+00 |
| Hotchkiss 13.2mm or Breda 31 | 5.85E+02 | 6.00E-01 | 2.00E+00 |
| M2HB of 1930s | 5.85E+02 | 6.00E-01 | 2.00E+00 |
| German MG34 w/ tripod | 5.85E+02 | 6.00E-01 | 3.00E+00 |
| German MG34 w/ bipod | 2.93E+02 | 3.00E-01 | 3.00E+00 |
| DShK38 | 4.88E+02 | 5.00E-01 | 2.00E+00 |
| German MG42 w/ tripod | 5.85E+02 | 6.00E-01 | 3.00E+00 |
| German MG42 w/ bipod | 2.93E+02 | 3.00E-01 | 3.00E+00 |
| Browning M1917 crew 3 | 2.93E+02 | 3.00E-01 | 3.00E+00 |
| Goryunov SG43 | 4.88E+02 | 5.00E-01 | 3.00E+00 |
| Degtiarev RPD M1927 | 1.95E+02 | 2.00E-01 | 4.00E+00 |
| FN MAG | 2.93E+02 | 3.00E-01 | 3.00E+00 |
| MG3 - bipod | 1.95E+02 | 2.00E-01 | 4.00E+00 |
| MG3 - tripod | 3.90E+02 | 2.00E-01 | 3.00E+00 |
| US M60 | 2.93E+02 | 3.00E-01 | 3.00E+00 |
| HK21 | 1.95E+02 | 2.00E-01 | 3.00E+00 |
| Soviet PK | 1.95E+02 | 2.00E-01 | 4.00E+00 |
| M2HB of 1970s | 2.93E+02 | 3.00E-01 | 2.00E+00 |
| Soviet NSV-12.7 | 1.95E+02 | 2.00E-01 | 2.00E+00 |
| STK 50MG | 1.95E+02 | 2.00E-01 | 2.00E+00 |
| Kord | 1.95E+02 | 2.00E-01 | 2.00E+00 |
| PKP Pecheneg | 1.95E+02 | 2.00E-01 | 4.00E+00 |
| **UTV** |  |  |  |
| Cub Cadet Challenger 550 | 1.17E+03 | 2.70E+01 | 6.56E+01 |
| Honda Pioneer 500 | 8.70E+02 | 3.10E+01 | 6.24E+01 |
| Can-Am Defender HD5 | 1.14E+03 | 3.80E+01 | 7.20E+01 |
| Polaris Ranger 500 | 9.40E+02 | 3.20E+01 | 7.36E+01 |
| Textron OffRoad | 1.31E+03 | 8.00E+01 | 1.02E+02 |
| Polaris General 100 EPS | 1.22E+03 | 1.00E+02 | 1.17E+02 |
| **Class 1-4 Trucks** |  |  |  |
| Ford T | 9.97E+02 | 2.00E+01 | 5.00E+01 |
| Land Rover | 3.10E+03 | 8.60E+01 | 1.04E+02 |
| Land Cruiser BJ45 | 2.48E+03 | 8.00E+01 | 1.04E+02 |
| Unimog 404 | 5.00E+03 | 1.10E+02 | 1.04E+02 |
| Ford F-350 Super Duty | 6.34E+03 | 3.00E+02 | 1.04E+02 |
| US Class 1 truck, light duty | 2.72E+03 | 1.00E+02 | 1.04E+02 |
| US Class 1 truck, heavy duty | 2.72E+03 | 2.10E+02 | 1.04E+02 |
| Chevrolet Colorado ZR2 | 2.76E+03 | 3.08E+02 | 1.04E+02 |
| Ford F-150 Platinum | 3.17E+03 | 3.95E+02 | 1.04E+02 |
| US Class 2a truck, light duty | 3.86E+03 | 2.36E+02 | 1.04E+02 |
| US Class 2a truck, heavy duty | 3.86E+03 | 3.81E+02 | 1.04E+02 |
| US Class 2b truck, light duty | 4.54E+03 | 2.35E+02 | 1.04E+02 |
| Chevrolet C-20 | 4.54E+03 | 1.15E+02 | 1.04E+02 |
| GMC Sierra 2500HD Denali | 4.53E+03 | 4.45E+02 | 1.04E+02 |
| US Class 2b truck, heavy duty | 4.54E+03 | 3.90E+02 | 1.04E+02 |
| Ram 3500 Limited | 5.57E+03 | 3.85E+02 | 1.04E+02 |
| US Class 3 truck, light duty | 6.35E+03 | 2.55E+02 | 1.04E+02 |
| US Class 3 truck, heavy duty | 6.35E+03 | 3.62E+02 | 1.04E+02 |
| US Class 4 truck, light duty | 7.26E+03 | 3.50E+02 | 1.04E+02 |
| US Class 4 truck, heavy duty | 7.26E+03 | 3.83E+02 | 1.04E+02 |
| **Technicals** |  |  |  |
| Ford T with Lewis MG | 9.97E+02 | 2.00E+01 | 2.00E+01 |
| Tachanka w/ PM1910 | 2.98E+03 | 1.34E+01 | 1.50E+01 |
| Chevrolet C-20 with ZPU-4 | 4.54E+03 | 1.15E+02 | 2.00E+01 |
| Land Rover w/ DShK | 3.10E+03 | 8.60E+01 | 2.00E+01 |
| Land Cruiser BJ45 w/ ZPU-2 | 2.48E+03 | 8.00E+01 | 2.00E+01 |
| Unimog 404 w/ ZPU-4 | 5.00E+03 | 1.10E+02 | 2.00E+01 |
| Unimog 404 w/ ZU-23-2 | 5.00E+03 | 1.10E+02 | 2.00E+01 |
| Ford F-350 Super Duty w/ ZPU-4 | 6.34E+03 | 3.00E+02 | 2.00E+01 |
| **Class 5-8 Trucks** |  |  |  |
| US Class 5 truck, light duty | 8.85E+03 | 2.88E+02 | 1.04E+02 |
| US Class 5 truck, heavy duty | 8.85E+03 | 3.85E+02 | 1.04E+02 |
| US Class 6 truck, light duty | 1.18E+04 | 2.60E+02 | 1.04E+02 |
| US Class 6 truck, heavy duty | 1.18E+04 | 3.62E+02 | 1.04E+02 |
| US Class 7 truck, light duty | 1.50E+04 | 3.30E+02 | 1.04E+02 |
| US Class 7 truck, heavy duty | 1.50E+04 | 3.50E+02 | 1.04E+02 |
| US Class 8 truck, light duty | 2.70E+04 | 3.30E+02 | 1.04E+02 |
| US Class 8 truck, heavy duty | 3.40E+04 | 4.75E+02 | 1.04E+02 |
| Cascadia tructor-trailer w/ DD15 engine | 3.60E+04 | 5.00E+02 | 1.04E+02 |
| **Towed guns** |  |  |  |
| Culverin Moyane 2-pdr | 3.40E+03 | 4.60E+00 | 5.00E+00 |
| Falcon 1-pdr | 2.45E+03 | 3.30E+00 | 5.00E+00 |
| Falconet 3/4-pdr | 1.70E+03 | 2.30E+00 | 5.00E+00 |
| early-to-mid-1600s saker | 7.14E+03 | 9.20E+00 | 5.00E+00 |
| early-to-mid-1600s minion | 5.18E+03 | 7.00E+00 | 5.00E+00 |
| early-to-mid-1600s falcon | 3.54E+03 | 4.60E+00 | 5.00E+00 |
| Saker in Battle of Cheriton | 5.70E+03 | 7.00E+00 | 5.00E+00 |
| Culverin 15-pdr | 7.01E+03 | 9.50E+00 | 5.00E+00 |
| Demi-culverin 9-pdr | 5.37E+03 | 7.30E+00 | 5.00E+00 |
| Saker 5-pdr | 3.67E+03 | 5.00E+00 | 5.00E+00 |
| Minion 4-pdr | 2.79E+03 | 3.80E+00 | 5.00E+00 |
| Falcon 2-pdr | 9.51E+02 | 1.30E+00 | 5.00E+00 |
| Falconet 1-pdr | 8.84E+02 | 1.20E+00 | 5.00E+00 |
| Robinet 3/4-pdr | 8.84E+02 | 1.20E+00 | 5.00E+00 |
| Saker | 4.42E+03 | 6.00E+00 | 5.00E+00 |
| Minion | 2.79E+03 | 3.80E+00 | 5.00E+00 |
| Falcon | 1.70E+03 | 2.30E+00 | 5.00E+00 |
| Falconet | 8.84E+02 | 1.20E+00 | 5.00E+00 |
| Robinet | 8.84E+02 | 1.20E+00 | 5.00E+00 |
| Regimental Gun of mid-1600s | 1.15E+03 | 1.30E+00 | 5.00E+00 |
| French Saker | 3.67E+03 | 5.00E+00 | 5.00E+00 |
| French Sixteenth | 2.52E+03 | 3.40E+00 | 5.00E+00 |
| French Thirty-second | 1.70E+03 | 2.30E+00 | 5.00E+00 |
| pre-Gribeauval, Austrian 12-pounder | 9.35E+03 | 1.15E+01 | 5.00E+00 |
| pre-Gribeauval, Austrian 12-pounder v2 | 9.35E+03 | 1.15E+01 | 5.00E+00 |
| Prussian 6-pdr (HA) | 5.24E+03 | 2.01E+01 | 1.50E+01 |
| Canon de 8 Gribeauval | 4.73E+03 | 5.30E+00 | 5.00E+00 |
| Light 6-pdr | 3.56E+03 | 5.00E+00 | 5.00E+00 |
| Canon de 8 Gribeauval (HA) | 9.91E+03 | 4.69E+01 | 1.50E+01 |
| Canon de 12 Gribeauval | 6.32E+03 | 7.50E+00 | 5.00E+00 |
| Canon de 12 Gribeauval (HA) | 1.15E+04 | 5.36E+01 | 1.50E+01 |
| Canon de 4 Gribeauval | 3.68E+03 | 4.80E+00 | 5.00E+00 |
| Canon de 4 Gribeauval (HA) | 8.86E+03 | 4.69E+01 | 1.50E+01 |
| British 1790 12-pdr | 6.71E+03 | 7.30E+00 | 5.00E+00 |
| British 1790 12-pdr (HA) | 1.19E+04 | 5.36E+01 | 1.50E+01 |
| British 1805 9-pounder | 6.20E+03 | 7.20E+00 | 5.00E+00 |
| British 1805 9-pounder (HA) | 1.14E+04 | 5.36E+01 | 1.50E+01 |
| French AnXI 12-pdr | 6.34E+03 | 7.30E+00 | 5.00E+00 |
| French AnXI 12-pdr (HA) | 1.15E+04 | 5.36E+01 | 1.50E+01 |
| French AnXI 6-pdr | 4.40E+03 | 5.00E+00 | 5.00E+00 |
| French AnXI 6-pdr (HA) | 9.58E+03 | 4.69E+01 | 1.50E+01 |
| Griffen 3-inch Ordnance Rifle | 4.24E+03 | 4.80E+00 | 5.00E+00 |
| Griffen 3-inch Ordnance Rifle (HA) | 9.42E+03 | 4.69E+01 | 1.50E+01 |
| Napoleon 1857 gun | 5.55E+03 | 6.80E+00 | 5.00E+00 |
| Napoleon 1857 gun (HA) | 1.07E+04 | 5.36E+01 | 1.50E+01 |
| Prussian C/61 | 5.50E+03 | 6.80E+00 | 5.00E+00 |
| Prussian C/61 (HA) | 1.07E+04 | 5.36E+01 | 1.50E+01 |
| RBL 12-pounder 8 cwt Armstrong gun | 3.61E+03 | 4.80E+00 | 5.00E+00 |
| RBL 12-pounder 8 cwt Armstrong gun (HA) | 8.79E+03 | 4.69E+01 | 1.50E+01 |
| Parrott 10 pdr rifle | 5.40E+03 | 8.80E+00 | 5.00E+00 |
| Parrott 10 pdr rifle (HA) | 1.06E+04 | 6.03E+01 | 1.50E+01 |
| German 96 m/A 77mm | 4.92E+03 | 6.60E+00 | 5.00E+00 |
| Canon de 75 modèle 1897 | 5.44E+03 | 6.60E+00 | 5.00E+00 |
| British QF 13-pounder, horse artil. | 8.43E+03 | 4.02E+01 | 1.50E+01 |
| British QF 13-pounder, field artil. | 6.00E+03 | 6.60E+00 | 5.00E+00 |
| 3.7 cm Pak 36 antitank gun | 3.57E+03 | 3.80E+01 | 1.50E+01 |
| 47mm APX antitank gun | 4.45E+03 | 3.80E+01 | 1.50E+01 |
| 5 cm Pak 38 (L/60) antitank gun | 4.17E+03 | 3.80E+01 | 1.50E+01 |
| 6 pdr atnitank towed gun | 4.64E+03 | 9.20E+01 | 1.50E+01 |
| 7.5 cm Pak 40 antitank gun | 9.63E+03 | 1.00E+02 | 2.00E+01 |
| 17 pdr antitank towed gun | 1.31E+04 | 1.47E+02 | 2.00E+01 |
| 17 pdr antitank towed gun, v.2 | 1.31E+04 | 1.47E+02 | 2.00E+01 |
| 8.8 cm Pak 43 | 1.69E+04 | 1.33E+02 | 2.00E+01 |
| 2A17 "D-30" in anti-tank role | 1.37E+04 | 2.00E+02 | 2.00E+01 |
| 100 mm anti-tank gun T-12 | 1.57E+04 | 2.40E+02 | 2.50E+01 |
| 2A36 “Giatzint-B” in anti-tank role | 2.60E+04 | 3.30E+02 | 2.00E+01 |
| 2A65 "Msta-B" | 1.61E+04 | 2.40E+02 | 2.00E+01 |
| 2A45M Sprut-B smoothbore 125mm antitank towed gun | 1.96E+04 | 2.40E+02 | 2.50E+01 |
| **AFVs and APCs** |  |  |  |
| Panhard 178, 1937 (wheeled) | 8.30E+03 | 1.80E+02 | 4.20E+01 |
| AAV-7, 1972 (tracked) | 2.28E+04 | 4.00E+02 | 1.35E+01 |
| Ratel, 1976 (wheeled) | 1.85E+04 | 2.82E+02 | 3.00E+01 |
| BVS 10, 2005 (tracked) | 1.06E+04 | 2.50E+02 | 1.50E+01 |
| BRM-1K (tracked) | 1.33E+04 | 3.00E+02 | 4.00E+01 |
| HJ-62C (tracked) | 1.36E+04 | 3.20E+02 | 4.00E+01 |
| Marder 1A3 (tracked) | 3.50E+04 | 5.00E+02 | 6.50E+01 |
| BMP-1P (tracked) | 1.34E+04 | 3.00E+02 | 4.50E+01 |
| BMP-2 (tracked) | 1.43E+04 | 4.00E+02 | 5.00E+01 |
| BMP-3M (tracked) | 1.87E+04 | 5.00E+02 | 4.50E+01 |
| Type 63 APC (tracked) | 1.26E+04 | 3.20E+02 | 4.00E+01 |
| BTR-60 (wheeled) | 1.03E+04 | 1.15E+02 | 6.00E+01 |
| BTR-80A (wheeled) | 1.46E+04 | 2.60E+02 | 6.00E+01 |
| BTR-82A (wheeled) | 1.60E+04 | 3.00E+02 | 4.00E+01 |
| M113A1 (tracked) | 1.12E+04 | 2.15E+02 | 3.50E+01 |
| MT-LB APC (tracked) | 1.19E+04 | 2.35E+02 | 3.00E+01 |
| S55 Mk1 car (wheeled) | 3.60E+03 | 1.14E+02 | 4.80E+01 |
| 1V13 (tracked) | 1.57E+04 | 2.40E+02 | 2.60E+01 |
| 1V110 (wheeled) | 3.60E+03 | 1.15E+02 | 3.50E+01 |
| **Self-propelled guns** |  |  |  |
| Marder III | 1.07E+04 | 1.48E+02 | 2.00E+01 |
| Sturmgeschütz III | 2.39E+04 | 2.96E+02 | 2.00E+01 |
| Jagdpanzer IV | 2.58E+04 | 2.96E+02 | 2.00E+01 |
| M36 Tank Destroyer | 2.86E+04 | 4.50E+02 | 2.50E+01 |
| SU-85 | 2.96E+04 | 4.93E+02 | 2.00E+01 |
| SU-100 | 3.16E+04 | 5.00E+02 | 2.00E+01 |
| BMPT Terminator | 4.70E+04 | 7.80E+02 | 4.50E+01 |
| 2S25 Sprut-SD | 1.80E+04 | 5.10E+02 | 4.50E+01 |
| **Tanks** |  |  |  |
| Mark IV | 3.20E+04 | 1.05E+02 | 5.00E+00 |
| Mark V Male, 1917 | 2.90E+04 | 1.50E+02 | 7.40E+00 |
| Whippet, 1917 | 1.27E+04 | 9.00E+01 | 1.29E+01 |
| FT tank | 6.50E+03 | 3.90E+01 | 5.00E+00 |
| FT-17 | 6.00E+03 | 3.90E+01 | 7.70E+00 |
| A7V | 3.30E+04 | 2.00E+02 | 7.00E+00 |
| Vickers 6-ton tank | 7.30E+03 | 9.80E+01 | 1.70E+01 |
| T-26 tank | 9.60E+03 | 9.00E+01 | 1.60E+01 |
| Char B1 bis | 2.80E+04 | 2.72E+02 | 2.10E+01 |
| SOMUA S35 | 1.92E+04 | 1.90E+02 | 1.60E+01 |
| Panzer 38(t) | 9.90E+03 | 1.50E+02 | 1.50E+01 |
| Mk III Valentine, 1939 | 1.70E+04 | 1.31E+02 | 1.29E+01 |
| Panzer III Ausf F, 1940 | 2.30E+04 | 3.00E+02 | 2.00E+01 |
| KV-1 tank | 4.50E+04 | 6.00E+02 | 1.60E+01 |
| T-34 of 1941 | 2.92E+04 | 5.00E+02 | 4.00E+01 |
| Mk VI Crusader I, 1941 | 1.97E+04 | 3.40E+02 | 2.40E+01 |
| Churchill Mk IV, 1941 | 3.90E+04 | 3.50E+02 | 1.30E+01 |
| M4 Sherman | 3.03E+04 | 3.50E+02 | 2.00E+01 |
| Tiger I | 5.70E+04 | 7.00E+02 | 2.00E+01 |
| T-34/85 | 3.20E+04 | 5.00E+02 | 2.00E+01 |
| Panzer IV | 2.50E+04 | 2.96E+02 | 1.60E+01 |
| IS-2 tank | 4.60E+04 | 6.00E+02 | 2.00E+01 |
| Sherman Firefly | 3.53E+04 | 4.25E+02 | 2.00E+01 |
| Panther | 4.48E+04 | 6.90E+02 | 3.00E+01 |
| Tiger II | 6.85E+04 | 7.00E+02 | 1.50E+01 |
| M26 Pershing | 4.17E+04 | 4.50E+02 | 8.00E+00 |
| Centurion tank | 5.20E+04 | 6.50E+02 | 1.70E+01 |
| T-54 | 3.60E+04 | 5.00E+02 | 3.50E+01 |
| M48 Patton | 4.50E+04 | 6.50E+02 | 2.10E+01 |
| M60 | 4.60E+04 | 7.50E+02 | 1.60E+01 |
| T-62 | 4.00E+04 | 5.80E+02 | 4.00E+01 |
| T-64 | 3.80E+04 | 7.00E+02 | 3.00E+01 |
| Chieftain tank | 5.60E+04 | 7.50E+02 | 3.00E+01 |
| Strv 103B (aka S-tank) | 3.97E+04 | 4.90E+02 | 3.00E+01 |
| T-72 tank | 4.25E+04 | 7.80E+02 | 4.50E+01 |
| T-80 | 4.60E+04 | 1.25E+03 | 4.80E+01 |
| M1 Abrams | 5.40E+04 | 1.50E+03 | 4.00E+01 |
| Challenger 1 | 7.00E+04 | 1.20E+03 | 3.00E+01 |
| M1A1 Abrams | 6.76E+04 | 1.50E+03 | 4.80E+01 |
| Challenger 2 | 7.50E+04 | 1.20E+03 | 4.00E+01 |
| Challenger 2, 1998 | 6.25E+04 | 1.20E+03 | 4.00E+01 |
| Leopard 2A6 | 6.23E+04 | 1.48E+03 | 4.80E+01 |
| Leopard 2A6, v2 | 6.23E+04 | 1.48E+03 | 4.80E+01 |
| Leopard 2A6, v3 | 6.23E+04 | 1.48E+03 | 4.80E+01 |
| M1A2 Abrams, 1996 | 6.20E+04 | 1.50E+03 | 5.47E+01 |
| T-14 Armata | 4.80E+04 | 1.50E+03 | 4.50E+01 |
| T-14 Armata v2 | 4.80E+04 | 1.50E+03 | 4.50E+01 |
| T-14 Armata v3 | 4.80E+04 | 1.50E+03 | 4.50E+01 |
| **Dump Trucks** |  |  |  |
| BELAZ 75710 | 8.10E+05 | 4.60E+03 | 6.40E+01 |
| Caterpillar 797F | 9.80E+05 | 4.00E+03 | 6.80E+01 |
| Liebherr T 282 | 6.20E+05 | 3.65E+03 | 6.40E+01 |
| John Deere 260E ADT | 4.67E+04 | 3.21E+02 | 6.40E+01 |
| John Deere 460E ADT | 7.40E+04 | 4.81E+02 | 6.40E+01 |
| Mack M917A3 | 4.28E+04 | 4.40E+02 | 6.40E+01 |
| Komatsu 980E-5 | 6.26E+05 | 3.35E+03 | 6.40E+01 |
| Caterpillar 789D | 3.04E+05 | 2.00E+03 | 5.70E+01 |
| Caterpillar 777G | 1.85E+05 | 1.03E+03 | 6.70E+01 |
| Rhino RT50 | 8.10E+04 | 5.25E+02 | 6.00E+01 |
| Bell B60E | 9.70E+04 | 5.77E+02 | 5.15E+01 |
| Bell B20E LGP | 2.64E+04 | 2.28E+02 | 4.67E+01 |
| Bergmann ADT-150 | 2.20E+04 | 1.62E+02 | 4.00E+01 |
| Caterpillar 775G | 1.28E+05 | 7.68E+02 | 6.80E+01 |
| Caterpillar 785D | 2.41E+05 | 1.45E+03 | 5.50E+01 |
| Hitachi EH4000AC-3 | 3.83E+05 | 2.37E+03 | 5.60E+01 |
| Hitachi EH5000AC-3 | 5.00E+05 | 2.64E+03 | 5.60E+01 |
| **Trains** |  |  |  |
| German trains, DB Class 103 loco | 5.16E+05 | 9.98E+03 | 2.00E+02 |
| Acela | 5.65E+05 | 1.24E+04 | 2.40E+02 |
| TGV Duplex | 4.24E+05 | 1.19E+04 | 3.20E+02 |
| UP coal train | 3.85E+07 | 2.61E+04 | 4.16E+01 |
| UP coal train | 3.85E+07 | 2.61E+04 | 9.60E+01 |
| BHP ore train | 9.97E+07 | 4.80E+04 | 2.70E+01 |
| **Other vehicles** |  |  |  |
| NASA Crawler, empty | 2.72E+06 | 5.50E+03 | 3.20E+00 |
| NASA Crawler, loaded | 8.15E+06 | 5.50E+03 | 1.60E+00 |

# **References for Supporting Information**

[1] Full, R. J. & Tu, M. S. Mechanics of six-legged runners. *J. Experi. Bio.* **148**, 129‒146 (1990).

[2] Full, R. J. & Tu, M. S. Mechanics of a rapid running insect: two-, four- and six-legged locomotion. J. Exp. Biol. **156**, 215‒231 (1991).

[3] Biancardi, C. M., Fabrica, C. G., Polero, P., Loss, J. F. & Minetti, A E. Biomechanics of octopedal locomotion: kinematic and kinetic analysis of the spider Grammostola mollicoma. *J. Experi. Bio.* **214**, 3433‒3442 (2011).

[4] Blickhan, R. & Full, R. J. Locomotion energetics of the ghost crab: II. Mechanics of the centre of mass during walking and running. *J. Experi. Bio.* **130**, 155‒174 (1987).

[5] Heglund, N. C., Cavagna, G. A. & Taylor, C. R. Energetics and mechanics of terrestrial locomotion. III. Energy changes of the centre of mass as a function of speed and body size in birds and mammals. *J. Experi. Bio.* **97**, 41‒56 (1982).

[6] Cavagna, G. A. & Kaneko, M. Mechanical work and efficiency in level walking and running. *J. Physiology* **268**, 467‒481 (1977).

[7] Taboga, P., Lazzer, S., Fessehatsion, R., Agosti, F., Sartorio, A., di Prampero, P. E. Energetics and mechanics of running men: the influence of body mass. *Eur. J. Appl. Physiol.* **112**; https://doi.org/10.1007/s00421-012-2389-6 (2012).

[8] Nudds, R.L., Codd, J.R. & Sellers, W. I. Evidence for a mass dependent step-change in the scaling of efficiency in terrestrial locomotion. *Plos One* **4**, e6927 (2009).

[9] Biewener, A. A. Muscle-tendon stresses and elastic energy storage during locomotion in the horse. *Comp. Biochem. Physiol. Part B: Biochem Mol. Bio.* **120**, 73‒87 (1998).

[10] Heglund, N. C., Fedak, M. A., Taylor, C. R. & Cavagna, G. A. Energetics and mechanics of terrestrial locomotion. IV. Total mechanical energy changes as a function of speed and body size in birds and mammals. *J. Experi. Bio.* **97**, 57‒66 (1982).

[11] Minetti, A. E., Ardigo, L.P., Reinach, E. & Saibene, F. The relationship between mechanical work and energy expenditure of locomotion in horses. *J. Experi. Bio.* **202**, 2329‒2338 (1999).

[12] Genin, J. J., P. A. Willems, G. A. Cavagna, R. Lair, and N. C. Heglund. "Biomechanics of locomotion in Asian elephants." *Journal of Experimental Biology* 213, no. 5 (2010): 694-706.

[13] Ahn, A. N., Furrow, E., & Biewener, A. A. (2004). Walking and running in the red-legged running frog, Kassina maculata. *Journal of Experimental Biology*, *207*(3), 399-410.

[14] Chen, J. J., Peattie, A. M., Autumn, K., & Full, R. J. (2006). Differential leg function in a sprawled-posture quadrupedal trotter. *Journal of Experimental Biology*, *209*(2), 249-259.

[15] Farley, C. T., & Ko, T. C. (1997). Mechanics of locomotion in lizards. *Journal of Experimental Biology*, *200*(16), 2177-2188.

[16] Griffin, T. M., & Kram, R. (2000). Penguin waddling is not wasteful. *Nature*, *408*(6815), 929-929.

[17] O’Neill, M. C., & Schmitt, D. (2012). The gaits of primates: center of mass mechanics in walking, cantering and galloping ring-tailed lemurs, Lemur catta. *Journal of Experimental Biology*, *215*(10), 1728-1739.

[18] Zani, P. A., Gottschall, J. S., & Kram, R. (2005). Giant Galapagos tortoises walk without inverted pendulum mechanical-energy exchange. *Journal of Experimental Biology*, *208*(8), 1489-1494.

[19] Kott, A., Gart, S. & Pusey, J. *A Dataset for Exploring Allometric Relations of Mass, Speed, and Power in Terrestrial Locomotion*, ARL-SR-0428. (US Army CCDC Army Research Laboratory, 2020). DOI: 10.13140/RG.2.2.14223.41126

[20] Randall-Reilly Construction. *Equipment World’s Spec Guide.* <https://www.specguideonline.com/> (2020).

[21] Kott, A. (2020). *An Extended Data Set for Explorations in Long-Range Forecasting of Military Technologies*. US Army CCDC Army Research Laboratory Adelphi United States.

[22] Whitt, R. F., Wilson, D. G., *Bicycling Science* (MIT Press, 1975).

[23] Sørensen, A., Tore Kristian Aune, Vegar Rangul, and Terje Dalen, (2018) The Validity of Functional Threshold Power and Maximal Oxygen Uptake for Cycling Performance in Moderately Trained Cyclists, *Sports,* **7**, 217

[24] Muetze, A. & Ying C. Ta, Electric Bicycles: A Performance Evaluation, *IEEE Industry Applications Magazine*, 2007, **13**(4), 12-21

[25] Juiced Bikes, “How far can I go on a single charge?”, undated, accessed 11/29/2020. url: <https://www.juicedbikes.com/pages/real-world-range-test>

[26] American Rails, Amtrak's Acela Express, <https://www.american-rails.com/acela.html>

[27] Trainweb, “Acela,” <http://www.trainweb.org/tgvpages/acela.html>

[28] Trainweb, “TGV Duplex,” <http://www.trainweb.org/tgvpages/duplex.html>

[29] Union Pacific, Union Pacific Railroad Increasing Train Speed on Rail Line in South-Central Arizona, January 21, 2015, <https://www.up.com/media/releases/0121_socen_az_speed.htm>

[30] Devereux, N. “End of the line for an icon: DB Class 103 Finale” *Railway Magazine*, July 10, 2018

[31] Guss, C., Union Pacific moves 'monster' loaded coal train with two distributed power sets to Wisconsin power plant, *Trains,* April 29, 2019, <https://trn.trains.com/news/news-wire/2019/04/29-union-pacific-moves-monster-loaded-coal-train-with-two-distributed-power-sets>

[32] Statista, Union Pacific's average train speed from FY 2013 to FY 2019, Jul 10, 2020, <https://www.statista.com/statistics/547745/average-train-speed-union-pacific-railroad/>

[33] Railway Gazette International, BHP breaks its own ’heaviest train’ record, 1 August 2001

[34] [Tegler](https://www.popularmechanics.com/author/7575/eric-tegler/), E., Launching To Space at a Crawl, Jul 16, 2019, Popular Mechanics, <https://www.popularmechanics.com/space/rockets/a15777930/launching-to-space-at-a-crawl/>

[35] Bejan, A. & Marden, J. H. Unifying constructal theory for scale effects in running, swimming and flying. *J. Experi. Bio.* **209**, 238‒248 (2006).

[36] McMahon, T. Size and shape in biology: elastic criteria impose limits on biological proportions, and consequently on metabolic rates. *Science* **179**, 1201‒1204 (1973).

[37] McMahon, T. A. Using body size to understand the structural design of animals: quadrupedal locomotion. *J. Appl. Physiol.* **39**, 619‒627 (1975).

[38] Biewener, A. A. & Taylor, C. R. Bone strain: a determinant of gait and speed?. *J Experi. Bio.* **123**, 383‒400 (1986).

[39] Biewener, A. A. Biomechanical consequences of scaling. *J. Experi. Bio.* **208**, 1665‒1676 (2005).

[40] Iriarte-Díaz, J. Differential scaling of locomotor performance in small and large terrestrial mammals, *J. Experi. Bio.* **205**, 2897–2908 (2002).

[41] Meyer-Vernet, N. & Rospars, J.-P. Maximum relative speeds of living organisms: Why do bacteria perform as fast as ostriches?. *Phys. Bio.* **13**, 066006 (2016).

[42] Marden, J. H. Scaling of maximum net force output by motors used for locomotion, <https://doi.org/10.1242/jeb.01484> (2005).

[43] Petrick, E. N., Janosi, Z. J. & Haley, P. W. *The Use of the NATO Reference Mobility Model in Military Vehicle Procurement*, no. 810373. (SAE Technical Paper, 1981).

[44] McCullough, M., Jayakumar, P., Dasch, J. & Gorsich, D. The next generation NATO reference mobility model development. *J. Terramechanics* **73**, 49‒60 (2017).

[45] Vong, T. T., Haas, G. A. & Henry, C. L. *NATO Reference Mobility Model (NRMM) Modeling of the DEMO III Experimental Unmanned, Ground Vehicle (XUV)*, ARL-MR-435. (US Army Research Laboratory, 1999).

[46] Boston Dynamics. *Spot Mini autonomous navigation*. <https://youtu.be/Ve9kWX_KXus> (2016 June 23).

[47] Ghost Robotics. *Ghost Vision 60*. <https://www.ghostrobotics.io/robots> (2019).

[48] Blickhan, R. The spring-mass model of bipedal locomotion on compliant legs. *J. Biomech.* **22**, 1217‒1227 (1989).

[49] Full, R. J. & Koditschek, D. E. Templates and anchors: neuromechanical hypotheses of legged locomotion on land. *J. Experi. Bio.* **202**, 3325‒3332 (1999).

[50] Seok, S. *et al.* Design principles for energy efficient legged locomotion and implementation on the MIT cheetah robot. *IEEE* *Trans. Mechatronics* **20**, 1117–1129 (2015).
