# Supplementary material for: From cockroaches to tanks: The same power-mass-speed relation describes both biological and artificial ground-mobile systems
Source: PLoS One. 2021 Apr 26;16(4):e0249066. doi: 10.1371/journal.pone.0249066 (PMC8075212; doi:10.1371/journal.pone.0249066)
Supplement: Attachment — Submitted filename: Response to Reviewers.docx [file pone.0249066.s002.docx]

The authors sincerely thank the reviewers for their insightful comments about the manuscript. We have carefully considered them and have made the changes detailed below to improve the clarity and accuracy of the manuscript.

**Reviewer #1:**

(1a) The paper investigates allometric relationship of mechanical power expended per unit of momentum (cost of transportation, CoT) for a wide range of natural and artificial systems exhibiting terrestrial locomotion. Authors find that the previously known relationship proposed for the mass range of 10^-3-10^1 kg by Heglund [13] holds over a much wider range of masses, 10^-3-10^4 kg, in animals and engineered systems alike. They also analyze the data available for engineered systems in the range of 10^4-10^6 kg and find that a qualitatively different scaling law applies for that range. Two linear functions describing CoT in the sub-ranges are fitted to the data. The resulting piecewise-linear relation holds for the whole range of masses thatwas considered (10^-3-10^6 kg). The relationship is additionally verified by checking that it holds on the data on sauropods and elephants that was not used in fitting of the empirical law, and finding that the data agrees with the law. Some existing ground-based robotic systems are considered in the context of the considered allometric relations and, for the most part, are found to be outliers. Implications of this fact for design of such systems are discussed.

(1b) All the claimed findings are, to the best of my knowledge, novel. While allometric studies that include both animals and engineered systems are not unprecedented (e.g. ref [39, 17] of the manuscript), no systematic studies of that sort were done before for terrestrial locomotion. Pre-existing work is properly cited.

(1c) Data collection methods used in the study are mostly sound. the only issue I found was the method in which the elephants and sauropods data was obtained. Mechanical power expended by the animals is derived from literature estimates of metabolic power by multiplying it by a constant factor. The factor is taken from the study on horses ([27], masses around 5e2 kg) and used for animals with masses at least two order of magnitude larger. Meanwhile, in Introduction the authors mention that the metabolic and mechanical cost of transportation scales differently. That makes the proportionate estimate dubious at best.

AUTHOR RESPONSE: Indeed, this is a valid concern. We address it as described below.

I ask that authors do one of the following:

1) provide a justification for proportional estimation of mechanical cost of transport from the metabolic cost, for the relevant mass range, or

2) provide a more careful estimation of mechanical cost of transport for elephants and sauropods and redo the related analysis and Figure 4, or

3) drop the elephant and sauropod data analysis from the paper altogether.

AUTHOR RESPONSE: We agree with the reviewer. To address this issue, we followed something of a hybrid of recommendations 1 and 2 above. Specifically, we provide a significant extension of the original discussion in Supplementary Information, in the section “Elephants and Sauropods”. There, we explain that our estimate of mechanical power is a likely lower limit on the actual (unknown) value, and show that a higher value would only improve the agreement between our formula and the data.

Another issue with this part of the study is that the data, while available in raw form in the primary sources, is processed to obtain the mechanical CoT estimates. It would be beneficial to make the processed data available.

AUTHOR RESPONSE: We agree. We added the processed data in Supplementary Information, in the section “Elephants and Sauropods”.

(1d) Also, Figure 4 is cluttered. Please only show the relevant range of masses and consider dropping the data for animals and artificial systems other than elephants and sauropods.

AUTHOR RESPONSE: We agree. We modified Figure 4 so that the data for elephants and sauropods are clearly visible.

This issue, however, is minor, as it the data is only used to provide additional support to a claim established with other data. Even if that part of the study is removed its primary claims can be sufficiently well supported.

AUTHOR’S RESPONSE: We agree.

(1e) Data analysis methods are sound. There is a minor presentation issue with ANCOVA analysis at the bottom of page 4: a single value is given as "difference" between slopes, and the value (0.964) is too large to be the true absolute difference between slopes that are close to 1. Please provide both of the slopes with confidence intervals.

AUTHOR RESPONSE: We apologize for the confusing wording. 0.964 is the mean of the biological and artificial data combined. ± 0.0224 is the difference between slopes of the biological and artificial data. We clarified this in the last two sentences of the last paragraph on page 4 by only reporting the magnitude of the difference between the slopes:

“We found no statistically significant difference between the intercepts (0.139; p-value = 0.0597) and only a small, although statistically significant, difference between the slopes (0.0448; p-value = 0.0313).”

(1f) Another minor issue encountered throughout the paper: values are reported to the third digit of the error margin. It is customary, at least in physical sciences, to round error margins down to one significant digit (two if the first one is 1) and also round the point estimate to the same decimal. For example, 0.964 \pm 0.0224 becomes 0.96 \pm 0.02. Similarly, for p-values usually only one significant digit is reported. Such conventions vary by the discipline, so I won't ask an adherence to this one, but in my opinion it could improve readability of the paper.

AUTHOR RESPONSE: We agree with the reviewer’s suggestion. Due to the change in reporting from comment (1e) above, we did not find any additional reported error margins to correct.

(1g) There is also a similar minor issue with the power estimate for EDMEC given in the Supplementary. The estimate is valid, but crude and must be treated as such. In light of that, the power consumption estimate of 4920W should be truncated to a single significant digit and presented as 5kW.

AUTHOR RESPONSE: Agreed. We changed 4920W to 5kW.

(1h) Language-wise, the manuscript is well-organized and accessible. There are a few places with typos and unclear language (listed below), but that is easily fixable and did not affect my ability to understand the intent of authors.

AUTHOR RESPONSE: We thank the authors for pointing these out. See responses below for corrections.

I recommend that the paper is accepted for publication after these issues are addressed.

----------------

Abstract:

"We show empirically not only..." - possible forgotten "that"

Fixed

Introduction:

"metabolic rate scales approximately to the 3/4 power of the organism's mass" - awkward wording

Revised to: “…the organism’s metabolic rate is approximately proportional to the organism’s mass to the 3/4 power…”

In the discussion of CoTm, a typo: "P/(M*V)" -> "Pm/(M*V)"

Corrected

Results:

p.10 last paragraph: "the slopes and intercepts of organism and system regression lines" - unclear

Revised to: “… compare the slope and intercept of the regression line fitted to the organism data to the slope and intercept of the regression line fitted to artificial system data …”

Supplementary:

The explanation of power expenditure section: "...is on the order of a magnitude or nearly constant magnitude of 10 body lengths per second..." - awkward

Revised to: "...is on the order of magnitude of 10 body lengths per second..."

Reviewer #2: The authors test how predictable the relationship between mass and mechanical power is for biologicaland artificial systems based on a previously proposed formula. The authors extend the formula to consider systems of larger mass, where the previously proposed formula failed. The authors then use this metric to analyze the efficiency of current artificial legged walkers and find that for the most part they are several times more inefficient than predicted by the formula. They discuss the reasons for this departure and argue for more biologically inspired technologies as a direction forward. The manuscript is straightforward, well written, and technically sound.

The authors sincerely thank the reviewer for the positive comments.
