# Supplementary material for: From cockroaches to tanks: The same power-mass-speed relation describes both biological and artificial ground-mobile systems
Source: PLoS One. 2021 Apr 26;16(4):e0249066. doi: 10.1371/journal.pone.0249066 (PMC8075212; doi:10.1371/journal.pone.0249066)
Supplement: Attachment — Submitted filename: Authors Responses to Reviewer 3.docx [file pone.0249066.s003.docx]

Authors Responses to Reviewer 3

**Reviewer #3:** The biggest challenges to this paper is in understanding the different metrics being used for ‘system/organism power’, perceiving whether these different metrics are reasonably comparable, and in interpreting the implications of the presented relationships. Comparing metrics…
It is reasonable to begin with the Heglund (Cavagna, Taylor, Fedak) studies of the 1980s for the broad-brush scaling data and exponent fits. Of the three I recall (metabolic, center of mass and center of mass + ‘internal’) the third is selected for the initial scaling equation (Heglund et al. 1982 IV). This is the energy put into the center of mass added to the energies of the limbs about the center of mass (with this metric, it is assumed there is no transfer between the two). However, it is the second that is used for the majority of the biological data survey. Why not use center of mass power for both (Heglund et al., 1982 III)?

**Authors’ Response**: The reviewer is bringing up an important point, and we accept that we owe the reader a more comprehensive explanation. As we stated in the beginning of the paper, our interest is in mechanical cost of transport, not metabolic, and as such, we do not use metabolic data. As far as measuring mechanical power expended by animals in their locomotion, several classes of technique are used and reported in literature: (a) force-measuring plates or treadmill, e.g., Arellano and Kram 2014 {see references at the end of this Response}, Biewener 1998; Biewener and Taylor 1986; Blickhan and Full 1987; Genin et al 2010; (b) video analysis of movement of body and limbs, e.g., Biancardi et al 2011; Cavagna and Kaneko 1977; Minetti et al 1999; and (c) the combination of the two. Other methods also exist, e.g. attaching inertial sensors to the body and limbs of an animal (e.g., Pfau et al 2006). We do use data obtained by any of these techniques, whichever is available in the literature. All of these techniques – with inevitable differences in assumptions and approximations – characterize the same fundamental quantity – the amount of mechanical energy expended to propel the system. The report (Kott et al 2020), see DOI: 10.13140/RG.2.2.14223.41126, describes our primary dataset and specifies for each data point, which paper was used as the source, and each source is clear as to which technique is used.

We also agree with the reviewer that different techniques are likely to produce somewhat different measures. However, we believe the differences are sufficiently small for an allometric study like ours, where the accuracy of the relations tends to be limited to an order of magnitude. In particular, the main difference is the contribution of the limb movements (i.e., the “internal” work), which tends to be on the order of 20-30% or less of the total work (e.g., Biancardi et al 2011, Minetti et al 1999) which makes no significant difference for an allometric study – e.g., mis-estimating the power by a factor of 1.5 would barely move the point on the allometric curve like our Fig.3.

We do agree with the reviewer, however, that we owe the reader a better explanation of the fact that a variety of different techniques are used to measure the expenditures of mechanical power for locomotion in animals. We address this by adding a discussion in Supplemental Information, section “Biological Organisms and sources of related data.”

**Reviewer #3:** Power proportional to mass and velocity (so a constant mechanical cost of transport): this appears to provide a better fit to the data too (Table 1: a=0; b=1; c=1?).

**Authors’ Response**: We agree that the suggestion of the reviewer P ~ M*V is a reasonable approximation for data with M<35,000 kg, producing a very simple formula, with only marginally worse fit than the coefficient values that we recommend in our paper. Specifically, the R2 for the reviewer suggested fit is 0.985 compared to 0.987 for the regression recommended in this study. We added a statement to that effect in the main text of the paper, at the end of the section “Results.”

**Reviewer #3:** A bigger issue is that neither form for mechanical power is (and is acknowledged to be) a very poor predictor for the metabolic or ‘biological engine’ power.

**Authors’ Response**: This is correct. However, we do not study metabolic power and make no mention of it, and we don’t use any of our findings to predict metabolic power.

**Reviewer #3:** But what is the ‘system’ power being described for the vehicles? It is presumably not the direct equivalent to the mechanical power demand of the animals – on flat level ground at constant speed this would be zero. Presumably it is the power supplied by the engine…? Under what condition? Maximum power or maximum speed?

**Authors’ Response**: We accept the reviewer’s criticism; we should have explained this matter more explicitly in the paper. Indeed we used engine power, and we used the rated power as by far the most readily and consistently available. Typically, in modern practice, ISO 1585 would be used to measure the rated power of engine. In older sources, we had to assume that the power quoted was comparable to modern rated power. Often, the actual mechanical power expended for locomotion, even at maximum rated speed and payload, would be lower than the rated power. However, as we mentioned earlier, differences are likely to be sufficiently small for an allometric study like ours, where the accuracy of the relations tends to be limited to an order of magnitude, and mis-estimating the power by, say, a factor of 1.5 would barely move the point on the allometric curve. We address the matter by adding a discussion in Supplemental Information, in section “Artificial systems and sources of related data.”

**Reviewer #3:** And then, what of the horse-drawn guns? Is the work calculated the ‘animal’ way or the ‘machine’ way?

**Authors’ Response**: We accept the reviewer’s criticism; we should have explained this matter more explicitly in the paper. In the case of horse-drawn guns, the only source of power are the horses. To approximate (again, we must remind that ours is an allometric study, and we seek mainly order-of-magnitude estimates) we used estimates as follows. Number of horses per gun were taken from various military history studies (references available); the power of a horse was taken from Minetti et al 1999 assuming 500 kg horse trotting at 15 km/hr. This is a lower limit of estimate because Minetti et all do not study a trained artillery horse that in addition to propelling itself, also pulls a wheeled gun, amounting typically to about 200-300 kg per horse. We added an explanation of this matter in Supplemental Information, in section “Artificial systems and sources of related data.”

**Reviewer #3:** And so it is not immediately clear to me that the chosen animal and machine powers are reasonably comparable. Implications…
If we believe there is some valid equivalency between the two metrics for power,..

**Authors’ Response**: We do believe that for all systems (biological or artificial) in our study we are looking at fundamentally the same metrics: the amount of mechanical energy expended to propel the system. Granted, the empirical or experimental methods of measuring this energy differ for different systems and by different experimenters. This is inevitable in a study like ours where we seek similarities across a very broad range of widely dissimilar systems. Nevertheless, they all refer to the same quantity: the amount of mechanical energy expended to propel the system.

We should add that there is a significant literature, from at least 1950s and until today, that includes comparisons of animal power and machine power, and inevitably uses values of power quantified by different methods for such widely different systems, e.g., Tucker 1975; Marden and Allen 2002; Isalgue 2008; Seok et al 2013; Trancossi 2015; Komoda and Wagatsuma 2017. In that respect, our paper is in line with precedents.

We agree with the author, however, that we owe to the reader a better explanation of the fact that a variety of different techniques are used to measure the expenditures of mechanical power. We addressed this by adding discussions in Supplemental Information, section “Biological Organisms and sources of related data” and section “Artificial systems and sources of related data.”

**Reviewer #3:** …the striking finding is the lack of evidence that a wheel improves matters.

**Authors’ Response**: The reviewer is asking a profound question. Indeed, should there be an expectation and a fundamental reason why the wheel (or track) improves energy efficiency of locomotion? Or perhaps, to the contrary, should the simplistic mechanical contraption called wheel (or track) perform worse than exquisitely perfected animal locomotion? These are questions that deserve future study and go beyond the scope of our paper.

**Reviewer #3:** If I were to start off with a one-human-power runner (measured the animal way), and then put the human mass on a bicycle, I would hope one human-power to drive either a much more massive load or to travel much more quickly.

**Authors’ Response**: Speaking specifically of a bicycle, indeed, in the literature, e.g., Wilson 1973, a bicycle has been noted for its exceptionally low energy cost of transportation. It is a known outlier, and Wilson 1973 attempts an explanation of why bicycle is such a remarkable outlier.

We accepted the reviewer suggestion and did add to our dataset several points corresponding to a bicycle. See more on this below.

**Reviewer #3:** That this is not evident across the data presented (compare 1000kg bull v.s truck) leads me to wonder whether 1) the two metrics for power are not comparable, or 2) trucks and tanks are horribly un-wheel-like.

**Authors’ Response**: As we discussed above, there is no obvious argument why any particular morphology, e.g., wheels, tracks, legs, etc., should be expected to be more beneficial than other in terms of cost of transportation. And also as we discussed above, the metrics for power are comparable: all of them are (inevitably approximate) quantifications of the mechanical power that the system (biological or artificial) expends to propel themselves.

**Reviewer #3:** Where does a motorbike on a road sit on this line? And a small train on a railway track? It would be nice to see where a couple of ‘good’ (fast or economical) wheeled vehicles sit on this plot.

**Authors’ Response**: We accept the reviewer’s recommendations. We have already included certain types of motorcycles such as UTVs in the set. We now added data points for human-powered bicycles, for electric bicycles, and for trains (both passenger and freight). We also checked for outliers: following Christensen 1997, we determined data to be an outlier if the standardized residual was greater than three scaled median absolute deviation (MAD) from the median standardized residual. Of the added data, the points for bicycles turned out as outliers – not surprisingly as we mentioned above.

We are particularly grateful to the reviewer for his suggestion regarding trains. We added data points for trains, and they fit well. Importantly, these data points extend significantly the overall range of mass and power in our dataset, by up to 2 orders of magnitude. See Fig. 3.

**Reviewer #3:** To offer ‘data on request’ or ‘see pdf’ is not very 2020. The data are indeed available online
<https://apps.dtic.mil/sti/pdfs/AD1098609.pdf>

**Authors’ Response**: We agree. We deleted “on request” and provided an easily available DOI: 10.13140/RG.2.2.14223.41126. On the reviewer’s suggestion, we also added the data as a table in Supplementary Information.

**Reviewer #3:** Which may bring into question the issue of novelty – that is a policy decision outside my remit

**Authors’ Response**: We don’t think publishing the data in a corporate report impacts the novelty of our paper. We leave this matter to the editor.

**Reviewer #3:** but why not provide them as a spreadsheet so that the reader can easily start trying out their own statistical approaches?

**Authors’ Response**: We agree. We added the data as a table in Supplementary Information.

**Reviewer #3:** The scaling relationship for the maximal speed in running animals falls over a bit at the larger sizes (Garland etc.) Please double check the values taken from Heglund – there may be an issue with conversion to SI units. See Blickhan, R. & Full, R. J. 1987 Locomotion energetics of the ghost crab II. Mechanics of the center of mass during walking and running. J. Exp. Biol. 130, 155-174.

**Authors’ Response**: We agree with reviewer. We are aware of all these papers, and we know about the reported issue with units’ conversion, and we did double-check the data.

**Reviewer #3:** It appears odd to resort to using metabolics to derive the elephant ‘animal-power’. Does not Genin, Willems, Cavagna, Lair and Heglund (J. Exp. Biol., 2010) provide a more appropriate starting point? It may be that the elephant data point would then fit rather poorly: note that Genin et al. report CoM energy fluctuations 1/3rd that of smaller mammals.

**Authors’ Response**: We are very grateful to the reviewer for pointing us to these data that previously escaped our attention. We now included them into the paper, and as the reviewer correctly suspected, the data fit poorly. In fact, Genin et al 2010 themselves suggested that the very low mechanical CoT they measured in their study is “The most striking finding…”. Apparently, no other animal is known to exhibit such a low mechanical CoT (at least “external” CoT). We added a note on this exceptional outlier in the paper’s “Discussion” session.

**Reviewer #3:** And including sauropods as empirical points does feel a bit of a stretch.

Authors’ Response: We agree, this is a stretch. To avoid any further controversy on this matter, we deleted the entire discussion and data of sauropods.

# References for the Response to Reviewer #3

Arellano, C. J. & Kram, R. Partitioning the metabolic cost of human running: a task-by-task approach. Integr. Comp. Bio. **54**, 1084–1098 https://doi.org/10.1093/icb/icu033 (2014).

Biewener, A. A. Muscle-tendon stresses and elastic energy storage during locomotion in the horse. *Comp. Biochem. Physiol. Part B: Biochem Mol. Bio.* **120**, 73‒87 (1998).

Biewener, A. A. & Taylor, C. R. Bone strain: a determinant of gait and speed?. *J Experi. Bio.* **123**, 383‒400 (1986).

Blickhan, R. & Full, R. J. Locomotion energetics of the ghost crab: II. Mechanics of the centre of mass during walking and running. *J. Experi. Bio.* **130**, 155‒174 (1987).

Biancardi, C. M., Fabrica, C. G., Polero, P., Loss, J. F. & Minetti, A E. Biomechanics of octopedal locomotion: kinematic and kinetic analysis of the spider Grammostola mollicoma. *J. Experi. Bio.* **214**, 3433‒3442 (2011).

Cavagna, G. A. & Kaneko, M. Mechanical work and efficiency in level walking and running. *J. Physiology* **268**, 467‒481 (1977).

Christensen, Ronald, “Residual Analysis and Outliers”, in Log-Linear Models and Logistic Regression, 2nd ed. New York, NY: Springer-Verlag, 1997

Genin, J. J., P. A. Willems, G. A. Cavagna, R. Lair, and N. C. Heglund. "Biomechanics of locomotion in Asian elephants." *Journal of Experimental Biology* 213, no. 5 (2010): 694-706.

Isalgue, A. On Scaling Laws and Maritime Transport. *J. Maritime Research* **5**, 33‒42 (2008).

Komoda, Kazuma, and Hiroaki Wagatsuma. "Energy-efficacy comparisons and multibody dynamics analyses of legged robots with different closed-loop mechanisms." Multibody System Dynamics 40, no. 2 (2017): 123-153.

Marden, J. H. & Allen, L. R. Molecules, muscles, and machines: universal performance characteristics of motors. *Proc. National Academy of Sciences* **99**, 4161‒4166 (2002).

Minetti, A. E., Ardigo, L.P., Reinach, E. & Saibene, F. The relationship between mechanical work and energy expenditure of locomotion in horses. *J. Experi. Bio.* **202**, 2329‒2338 (1999).

Pfau, Thilo, Thomas H. Witte, and Alan M. Wilson. "Centre of mass movement and mechanical energy fluctuation during gallop locomotion in the Thoroughbred racehorse." *Journal of Experimental Biology* 209, no. 19 (2006): 3742-3757.

Seok, S. *et al.* Design principles for energy efficient legged locomotion and implementation on the MIT cheetah robot. *IEEE* *Trans. Mechatronics* **20**, 1117–1129 (2015).

Trancossi, M. What price of speed? A critical revision through constructal optimization of transport modes. *Intl. J. Energy Environ. Engineer.* **7**, 425‒448 (2016).

Tucker, V. A. The energetic cost of moving about. *American Scientist*. **63,** 413–419 (1975).

Wilson, S. S., Bicycle Technology, Scientific American, Vol. 228, No. 3 (March 1973), pp. 81-91
