# Supplementary material for: From cockroaches to tanks: The same power-mass-speed relation describes both biological and artificial ground-mobile systems
Source: PLoS One. 2021 Apr 26;16(4):e0249066. doi: 10.1371/journal.pone.0249066 (PMC8075212; doi:10.1371/journal.pone.0249066)
Supplement: Attachment — Submitted filename: Authors Responses to Reviewer 3 for Rev5.docx [file pone.0249066.s004.docx]

Authors Responses to Reviewer 3

**Reviewer #3:**

The major issue with this paper is that it assumes comparability between two potentially quite different forms of power. The vehicle values are for engine power, if possible, the ‘rated power’ (responses). This is the power (torque x angular velocity) at the end of the drive shaft. The rated power is useful in informing (once other losses are considered, and given suitable gearing) the capacity of a vehicle to accelerate, pull a load up an incline, or overcome drag.
The animal values are the rate of mechanical work of the center of mass during steady state locomotion.
Is it reasonable to assert an equivalency between ‘rated power’ and ‘center of mass power’? The difficulty is that the ‘center of mass power’ of a wheeled vehicle at any steady speed (even exceedingly high, and against drag) on level ground is zero. So ‘rated power’ cannot sensibly translate to ‘center of mass power’. But is the reverse likely? Does the center of mass power of an animal display something equivalent to its rated power? One can certainly imagine cases where this fails (consider cycling), but might it be reasonable for legged locomotion?
For the purposes of this paper, it would appear sufficient to state it as an assumption that it is reasonable, with whatever justification can be thought of and while acknowledging that others have also made this assumption.
At the moment, this is not sufficiently addressed. The term ‘rated power’ only appears in the responses – a description of the vehicle power close to the one I give above is required. Also in the responses is the justification:
“We do believe that for all systems (biological or artificial) in our study we are looking at fundamentally the same metrics: the amount of mechanical energy expended to propel the system.”
This is insufficient. Why is the center of mass power (that can be zero given wheels) ‘the amount of mechanical energy expended to propel the system’. Until an explicit work-around is given for this, any informed reader will view this study as a comparison of apples and oranges.

**Authors’ Response**: Upon further pondering the issue brought up by the reviewer, we have to accept that the reviewer has a strong point. Yes, the two quantities are to a large extent “apples and oranges.” We conclude that the mechanical power output of a vehicle’s engine is indeed conceptually different from the “external work” of an animal locomotion as typically measured by experimental biologists using the ground reaction forces procedure of Cavagna 1975 [85] (all numbers in square brackets refer to the references in the main manuscript) or similar procedure. However, using the approach of Cavagna et al 1977 [88] we also found a way to demonstrate that the “external work” per unit time in a steady state locomotion of an animal can serve as an order-of-magnitude approximation of the mechanical power output of the animal’s muscles, which is conceptually comparable to the mechanical power output of a vehicle’s engine.

Let’s discuss this in some detail.

First, what is the mechanical power that we are exploring?

In the case of vehicles, we take the mechanical power output of a vehicle’s engine *P_e_* as approximately equal (for the purposes of our allometric, order-of-magnitude study) to the mechanical power expended for the vehicle’s locomotion. In doing so we neglect the energy that a vehicle may expend while stationary, e.g., for operating air conditioning, as well as the expenditures of mechanical energy for maintaining the operation of the engine itself, e.g., tanks’ cooling fans that may take 10-15% of the engine output [83, p.258]. In steady state locomotion, at constant altitude, the entire mechanical power output of the engine *P_e_* is transformed eventually into heat and transferred to the environment of the vehicle (air and ground). This involves a complex chain of energy transformations ultimately ending in dissipative processes such as friction of sliding elements, gears, joints, and hysteresis in the rubber of tires [83, p.228; 84].

Similarly, in the case of animals, we take the net mechanical power output of an animal’s muscles *P_a_* as approximately equal the mechanical power expended for the animal’s locomotion. In doing so we neglect the mechanical energy that an animal may expend while stationary, or any mechanical energy that the animal might use (while moving) for purposes other than locomotion. In steady state locomotion, at constant altitude, the entire mechanical power output of the muscles *P_a_* is transformed eventually into heat and transferred to the environment of the animal (air and ground). This involves a complex chain of energy transformations (potential to kinetic, and back, as well as elastic, etc.) ultimately ending in dissipative, heat-generating processes such as inelastic deformations of tendons and muscles [85; 86; 87].

Thus, if we could use data for *P_e_* for vehicles, and *P_a_* for animals, we would have conceptually comparable, “apples to apples” quantities.

However, that’s where we face a major problem: the data for *P_a_* are not available. The conceptual similarity of *P_e_* and *P_a_*  breaks down when we turn to the ways in which the mechanical power output is experimentally measured, and the corresponding availability of experimental data. In vehicles, the mechanical power output of an engine *P_e_* can be measured directly in several relatively simple ways, typically involving measuring the torque and angular velocity at the end of the engine’s shaft, and for vehicles the data are usually available at least for the maximum “rated” power output (see [89]) approximately corresponding to the vehicle travelling at maximum rated speed and maximum load weight. In animals, on the other hand, measuring the rate at which muscles produce and output mechanical energy *P_a_* is extremely difficult [85] and the corresponding data are essentially unavailable.

Instead of *P_a_*, experimental biologists measure so called external work of an animal, the rate of which (work per unit time) we designate here as *P_ext._*  This quantity in locomotion of animal is derived using a number of approaches. Most of them assess the changes in the body’s potential and kinetic energy. The movement of the body’s center of mass can be determined either using a video recording of the movement of a marker placed near the center of mass, or most commonly from the forces that the body exerts against the ground (or force plates), i.e., the ground reaction forces, often using the procedure popularized by Cavagna 1975 [85].

For the purposes of our research, the challenge is that *P_ext_*  is not directly comparable to *P_a_* . One major difference between *P_ext_* and *P_a_* is that *P_ext_* includes the elastic energy stored in muscles and tendons of an animal [88] and as such can be significantly greater than *P_a_* . However, we show that although *P_ext_* and *P_a_* are fundamentally different quantities, *P_ext_* can serve as an order-of-magnitude approximation of *P_a_* .

Let’s derive this.

In the following, we build on the approach and data of Cavagna and co-workers 1977 [88]. Per Eqs. 5 and 6 of [88], neglecting the negative work of muscles (as [88] does), the efficiency γ with which muscles transform chemical energy into positive work is *γ = P_a_/P_met_ = (P_ext_ + P_int_ – P_elast_)/P_met_*, where *P_a_* and *P_ext_* were introduced earlier, *P_int_* is the rate of work associated with movements of an animal’s limbs with respect to its center of gravity, *P_elast_* is the power recycled via elastic storage within an animal’s limbs, and *P_met_* is the chemical power input to muscles. Per [88], *γ* ranges from 0.2 to 0.3, and empirical values of *α = P_ext_/P_met_* range from 0.15 to 0.75, for a number of species (rhea, turkey, spring hare, kangaroo, dog and monkey).

Then, rearranging, the mechanical power output produced by muscles *P_a_ = γ·P_met_ = P_ext_ + P_int_ – P_elast_ = (γ/α)·P_ext_*. Taking the range of values of γ and α mentioned above, we find that the values of *P_a_/P_ext_ = γ/α* are in the range 0.27 -- 2.0.

Therefore, although *P_ext_* and *P_a_* are not directly comparable, *P_ext_* can serve as an order-of-magnitude approximation of *P_a_*. Although the range of values above suggest that *P_ext_* could be twice smaller or nearly 4 times larger than *P_a_*, this is acceptable for our allometric, order-of-magnitude study. For example, to become an outlier in Fig. 3 (see the discussion of outliers in the Discussion section of the main manuscript), a data point would have to disagree with the predicted value of power by a factor of approximately 5. And although the values of *α* provided in [88] cover only 6 species, Fig. 3 illustrates that data for many other species fall into the same pattern, consistent with the premise that *P_ext_* can serve as an order-of-magnitude approximation of *P_a_*.

To reflect all of the above, we completely re-wrote the “Data” section of the manuscript, and made a major addition to the “Discussion” section.

**Reviewer #3:** 
I wonder whether the biological literature is as exhaustive as claimed. I would suggest doing a citation search on Cavagna, 1975 ‘force platforms as ergometers’. Off the top of my head, are the reported values for cats, tortoise, penguins all unusable? Any lizards?

**Authors’ Response**:

We are grateful for the reviewer’s recommendation. We did find additional useable data for frogs, geckos, other lizards, penguin, lemurs, and giant tortoises. All of these data, with the exception of only one of the gecko’s points, agree well with the Fig. 3, with no changes to the correlation coefficients. The data were included in the paper.

Regarding cats, a number of studies exist, going back to Manter 1938, and more recent Bishop, Pai and Schmitt 2008, and Granatosky et al 2018. However, we were unable to extract from them data useable for our purposes.

On the other end of the power spectrum, we found and added data for an enormous vehicle called NASA Crawler. In spite of its uniqueness, it also fits well.

We made corresponding additions to the manuscript and to the Supplemental Information.
